# Anemia Associated with Worse Outcome in Diffuse Large B-Cell Lymphoma Patients: A Single-Center Retrospective Study

**DOI:** 10.4274/tjh.2017.0437

**Published:** 2018-08-05

**Authors:** Kenji Matsumoto, Shin Fujisawa, Taiki Ando, Megumi Koyama, Satoshi Koyama, Yoshimi Ishii, Ayumi Numata, Wataru Yamamoto, Kenji Motohashi, Maki Hagihara, Hideaki Nakajima

**Affiliations:** 1 Yokohama City University Faculty of Medicine, Department of Hematology and Clinical Immunology, Yokohama, Japan; 2 Yokohama City University Faculty of Medicine, Department of Hematology, Yokohama, Japan; 3 Kanagawa Cancer Center, Clinic of Medical Oncology, Yokohama, Japan; 4 Kanagawa Cancer Center, Clinic of Hematology, Yokohama, Japan

**Keywords:** Anemia, Diffuse large B-cell lymphoma, Hemoglobin

## Abstract

**Objective:**

Useful prognostic biomarkers for diffuse large B-cell lymphoma (DLBCL) patients have been reported. To determine the prognostic value of hemoglobin (Hb) level in DLBCL patients, we performed a retrospective study.

**Materials and Methods:**

We evaluated disease outcome, progression-free survival (PFS), overall survival as the endpoint, and clinical and laboratory factors affecting the outcome of 185 DLBCL patients who had received rituximab, cyclophosphamide, doxorubicin, vincristine, and prednisolone therapy during 2004-2014.

**Results:**

The study group included 121 men and 64 women with a median age of 66 years minimum-maximum: 21-83 years. In univariate analysis, factors independently associated with worse PFS were Eastern Cooperative Oncology Group performance status ≥2, Ann Arbor stage III or IV, anemia with Hb levels of <10 g/dL, and serum albumin of <3.5 g/dL. In multivariate analysis, anemia with Hb levels of <10 g/dL and Ann Arbor stage III or IV were found to be international index-independent prognostic factors (hazard ratio: 2.4; p=0.04).

**Conclusion:**

Anemia is an independent prognostic marker of poor outcome in DLBCL patients. Hb can be an easily available prognostic marker for risk stratification in these patients.

## Abstract (tr)

**Amaç:**

Diffüz büyük B hücreli lenfoma (DBBHL) hastaları için yararlı prognostik belirteçler bildirilmiştir. DBBHL hastalarında hemoglobin (Hb) düzeyleri ile prognoz ilişkisini araştırmak amacıyla retrospektif bir çalışma yaptık.

**Gereç ve Yöntemler:**

2004-2014 yılları arasında rituksimab, siklofosfamid, doksorubisin, vinkristin ve prednizolon tedavisi almış olan 185 DBBHL hastasının klinik ve laboratuvar bulguları değerlendirildi ve bunların hastalık sonuçlarına, ilerlemesiz sağkalım (PFS) ve toplam sağkalıma (TS) etkileri araştırıldı.

**Bulgular:**

Araştırma grubunda 121 erkek ve 64 kadın vardı, ortanca yaş 66 yıldı (minimum-maksimum: 21-83 yıl). Tek değişkenli analizde Eastern Cooperative Oncology Group performans durumunun ≥2 olması, Ann Arbor evre III veya IV olması ve Hb değerinin <10 g/dL ile anemi olması PFS ve TS’yi etkileyen bağımsız değişkenler olarak bulundu. Çok değişkenli analizde ise Hb değerinin <10 g/dL ile anemi ve Ann Arbor evre III veya IV olması uluslararası indeks-bağımsız prognostik faktörler olarak bulundu (Hazard ratio: 2,4; p=0,04).

**Sonuç:**

Anemi, DLBCL hastalarında kötü sonuçların bağımsız prognostik bir göstergesidir. Hb, bu hastalarda risk sınıflandırması için kolayca bulunabilen bir prognostik belirteç olabilir.

## Introduction

Diffuse large B-cell lymphoma (DLBCL) is the most common non-Hodgkin lymphoma subtype, which accounts for 30%-40% of newly diagnosed malignant lymphoma cases [1]. Standard immunochemotherapy, such as R-CHOP containing rituximab, cyclophosphamide, hydroxydaunorubicin, vincristine, and prednisolone, has been proven to be beneficial for the outcome of DLBCL patients; however, approximately one-third of patients with advanced-stage disease still experience relapse or are refractory to therapy [2,3]. There are many well-known established prognostic models to identify patients at high risk of disease progression or relapse, or refractory to therapy, because it is an urgent necessity to improve outcomes. The International Prognostic Index (IPI), a well-known and useful tool for predicting the clinical outcomes of DLBCL patients, includes age, serum lactate dehydrogenase (LDH) level, performance status, Ann Arbor stage, and number of extranodal lesions [4]. Variants are reported for elderly patients (age-adjusted IPI) and patients treated with rituximab (R-IPI) [5]. Recently, the National Comprehensive Cancer Network (NCCN) published a reformed IPI, which weighted the scoring for increasing age and LDH levels [6]. Unfortunately, clinical risk stratification models, such as IPI, R-IPI, and NCCN-IPI, are not completely accurate in identifying patients who will not be sufficiently cured by first-line R-CHOP therapy. There is an urgent necessity for new prognostic biomarkers. In particular, anemia is commonly associated with lymphoma, even in chemotherapy-naive patients, and in the absence of bone marrow (BM) involvement [7]. The pathogenesis of lymphoma-associated anemia is multifactorial and may include BM dysfunction, problems with iron reutilization, and an inadequate erythropoietin response. In this study, we evaluated the significance of pretreatment hemoglobin (Hb) levels.

## Materials and Methods

This study was approved by the Yokohama City University Medical Center Clinical Research Ethics Board. All procedures used in this study were in accordance with the Declaration of Helsinki.

### Patients

We reviewed the records of 226 patients diagnosed with DLBCL at Yokohama City University Medical Center during 2004-2014. Forty-one patients were excluded due to the following reasons: transfer to another hospital after diagnosis (n=12), other regimens (n=18), radiation only (n=5), received supportive care only because of poor performance status (n=3), double cancer (n=1), early death within 30 days (n=1), and lack of information (n=1). The following clinical data on 185 patients were collected from medical records and pathology reports: histological confirmation of diagnosis, sex, age, Ann Arbor clinical stage, presence of B symptoms, LDH levels, serum albumin levels, Eastern Cooperative Oncology Group (ECOG) performance status, and BM involvement data. Hematology data, including full blood count, were obtained at diagnosis, 1-7 days before initiating treatment. The institutional lower limit of normal (LLN) of hemoglobin (Hb) in complete blood count was set as 13.8 g/dL for male patients and 11.3 g/dL for female patients. Severity of anemia was graded by Hb levels according to the National Cancer Institute Common Terminology Criteria for Adverse Events, version 4.0, as follows: G1, <LLN to 10.0 g/dL; G2, <10.0 to ≤8.0 g/dL; G3, <8.0 g/dL or transfusion indicated; and G4, life-threatening. BM involvement was detected by either aspiration or biopsy of BM, except for positron emission tomography imaging.

Patients were treated with a standard immunochemotherapy regimen containing rituximab, cyclophosphamide, doxorubicin, vincristine, and prednisolone (R-CHOP). The R-CHOP regimen comprised 6-8 cycles of 750 mg/m^2 ^cyclophosphamide, 50 mg/m^2 ^doxorubicin, and 1.4 mg/m^2 ^(maximum 2.0 mg/kg body weight) vincristine on day 1; 100 mg/body prednisolone on days 1-5; and 375 mg/m^2 ^rituximab per cycle for 21 days.

Progression-free survival (PFS) was defined as the time from R-CHOP therapy initiation to lymphoma progression, death from any cause, or last follow-up. Overall survival (OS) was defined as the time from diagnosis to death from any cause or until the time of last follow-up for patients who remained alive.

### Statistical Analysis

Kaplan-Meier analysis was used to calculate PFS. The log-rank test was used to assess univariate associations between PFS and prognostic variables. A forward-backward stepwise variable selection for the Cox proportional hazards model was used for multivariate analysis. A value of p<0.05 was considered statistically significant. All statistical analyses were performed with EZR (version 1.10) [8], which is a graphical user interface for R (R Foundation for Statistical Computing, version 2.13.0), using the modified version of the R commander (version 1.6-3) designed to add statistical functions frequently used in biostatistics.

## Results

### Patient Characteristics

Among the 226 patients diagnosed with DLBCL at Yokohama City University Faculty of Medicine during 2004-2014, 185 patients satisfied the inclusion criteria. The characteristics of the study participants are summarized in Table 1. The study included 121 males and 64 females, with a median age of 66 years (minimum-maximum: 21-83 years).

### Patient Outcomes and Prognostic Factors

**Figure 1:**
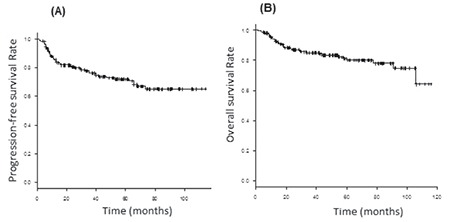
A) Progression-free survival (PFS) and B) overall survival (OS) in 185 patients with diffuse large B-cell lymphoma. The median observation period in surviving patients was 55 months (minimum-maximum: 4.8-117 months). The 3-year PFS rate was 76.1% and the 3-year OS rate was 80.1%.

The median observation period in surviving patients was 55.3 months (minimum-maximum: 4.8-117 months). The estimated PFS rate of all patients at 3 and 5 years was 76.1% and 72.0%, respectively. The OS rate at 3 and 5 years was 85.0% and 80.1%, respectively (Figure 1).

**Figure 2:**
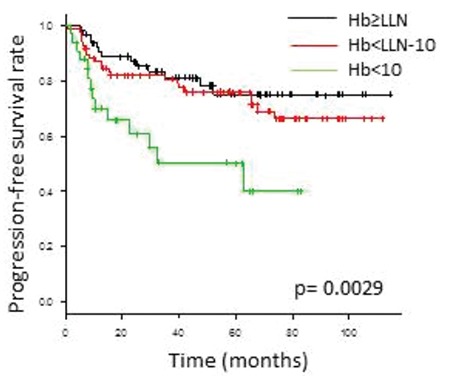
Analysis of the impact of anemia on treatment outcomes in patients with diffuse large B-cell lymphoma treated with rituximab, cyclophosphamide, doxorubicin, vincristine, and prednisolone therapy (n=185). Kaplan-Meier plots for progressionfree survival according to the grade of baseline anemia. Hb: Hemoglobin, LLN: Lower limit of normal.

The mean baseline Hb (±standard deviation) was 12.1±2.2 g/dL in male patients and 11.4±1.8 g/dL in female patients. Eight-seven patients (47%) had G≥1 and 33 patients (18%) had G≥2 baseline anemia. Patients with G≥2 anemia showed inferior PFS compared with those with no (G0) or G1 anemia (p<0.0029; Figure 2).

On univariate analysis, factors associated with worse PFS included ECOG performance status ≥2 (vs. ≤1: p=0.041), Ann Arbor clinical stage (CS) ≥3 (vs. ≤2: p<0.001), G≥2 anemia (vs. G≤1: p=0.001), serum albumin <3.5 g/dL (vs. ≥3.5 g/dL: p=0.008), and BM involvement (vs. negative: p<0.001).

**Figure 3:**
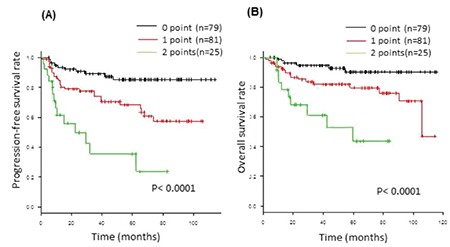
Progression-free survival and B) overall survival in 185 patients with diffuse large B-cell lymphoma according to prognostic index. Kaplan-Meier plots for event-free survival according to the grade of baseline anemia and clinical stage.

Multivariate analysis showed that Ann Arbor CS ≥3 [hazard risk (HR): 3.0; 95% confidence interval (CI): 1.4-6.4; p=0.005), G≥2 anemia (HR: 2.3; 95% CI: 1.2-4.3; p=0.012), and BM involvement (HR: 1.9; 95% CI: 1.0-3.6; p=0.037) were identified as risk factors (Table 2). Because the CS IV criteria include BM involvement, CS≥3 and G≥2 anemia remained as independent determinants. These factors were each assigned a score and the sum was tested as a prognostic index for PFS. The 3-year PFS of patients on an R-CHOP regimen with score 0 (n=79), score 1 (n=81), and score 2 (n=27) was 89.1%, 73.9%, and 35.5%, respectively (p<0.001), and the 3-year OS of patients on an R-CHOP regimen with score 0 (n=79), score 1 (n=81), and score 2 (n=27) was 94.6%, 82.0%, and 61.4%, respectively (p<0.001; Figures 3A and 3B).

## Discussion

Anemia is commonly encountered in patients with malignant lymphoma or lymphoproliferative disorders. The incidence was reported as approximately 39% previously [7]. The purpose of the current study was to determine whether anemia has a prognostic value in DLBCL, for which a number of prognostic tools have recently been devised. Because Hb status is a standard laboratory parameter, easy to measure, cheap, and highly reproducible in the clinical setting, Hb level could be readily incorporated into a newly differentiated prognostic index.

In follicular lymphoma, Hb of <12 g/dL was known to be prognostic [9,10], but Chen et al. [11] showed that Hb of <12 g/dL did not show a significant association with inferior PFS or OS in DLBCL patients treated with rituximab-containing immunochemotherapy. In a recent study by Hong et al. [12], G>2 anemia showed an association with inferior event-free survival , which was the same as our result.

Baseline anemia may not be an independent factor but rather a consequence of bone marrow involvement, which in turn may actually just be a part of various factors causing anemia. Tisi et al. [13] showed that lymphomatous BM involvement is independent of the occurrence of anemia, with no difference in Hb levels observed according to the BM status (median: 11.8 g/dL for patients without BM infiltration vs. 10.9 g/dL for those with BM infiltration, p=0.27). They also concluded that an elevated level of interleukin-6, a proinflammatory cytokine, was the dominant factor affecting anemia, and reduced erythropoietin synthesis may result in anemia.

### Study Limitations

This study had some limitations, including its observational retrospective design and the analysis of a small number of patients.

## Conclusion

In conclusion, anemia assessed by pretreatment Hb of <10.0 g/dL was an overall prognostic factor, and Hb is very easy to analyze in clinical settings, with almost no additional cost. For patients with DLBCL treated with R-CHOP, our new prognostic index, which consists of Hb and clinical stages, may be helpful for selecting the treatment strategy, including investigational salvage therapy, although the effectiveness of our index should be validated in a larger cohort.

## Figures and Tables

**Table 1:**
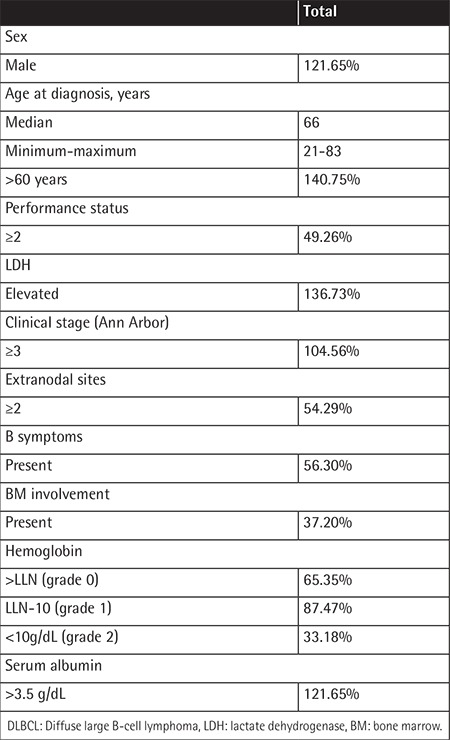
Clinicopathological characteristics of patients with diffuse large B-cell lymphoma.

**Table 2:**
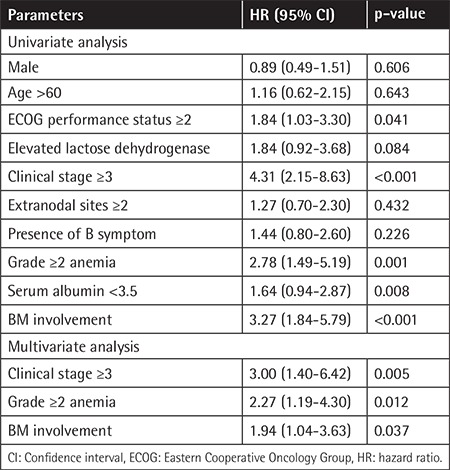
Univariate and multivariate analysis for progressionfree survival in all patients with diffuse B-cell lymphoma (n=185).
